# Telemonitored Human Circadian Temperature Dynamics During Daily Routine

**DOI:** 10.3389/fphys.2021.659973

**Published:** 2021-05-10

**Authors:** Qi Huang, Sandra Komarzynski, Matei Bolborea, Barbel Finkenstädt, Francis Albert Lévi

**Affiliations:** ^1^Cancer Chronotherapy Team, Warwick Medical School, Coventry, United Kingdom; ^2^Department of Statistics, University of Warwick, Coventry, United Kingdom; ^3^School of Life Sciences, University of Warwick, Coventry, United Kingdom; ^4^UPR “Chronotherapy, Cancers and Transplantation”, Faculty of Medicine, Paris-Saclay University, Villejuif, France; ^5^Hepato-Biliary Center, Paul-Brousse Hospital, Assistance Publique-Hôpitaux de Paris, Villejuif, France

**Keywords:** circadian rhythm, temperature, age, sex, telemedicine, inter-individual variability, telemonitoring chronotherapy

## Abstract

**Background:**

Circadian rhythms in body temperature coordinate peripheral molecular clocks, hence they could potentially predict optimal treatment timing (chronotherapy) in individual patients. Circadian parameters in chest surface body temperature (Chesttemp) were recorded remotely and in real time through the use of wearable sensors.

**Methods:**

The dynamics of circadian oscillations in Chesttemp and core body temperature (Coretemp) and their moderation by sex and age were analysed in 38 men and 50 women, aged 21–78 years. In two studies (ST1 and ST2), Chesttemp was measured every minute and teletransmitted using a BLE-connected sensor for 3.6–28.3 days. Additionally, in ST2, Coretemp was recorded per minute in 33 age- and sex-stratified subjects using electronic ingestible pills with radio-frequency transmissions. Circadian parameters were computed using spectral analysis and cosinor modelling. The temporal relations between Chesttemp and Coretemp cosinor parameters were summarised with principal component (PC) analysis. The effect of sex and age was analysed through multivariate regression.

**Results:**

Using spectral analysis, a dominant period of 24- or 12-h was identified in 93.2% of the Chesttemp and in 100% of the Coretemp time series. The circadian parameters varied largely between-subjects both for Chesttemp (ranges: mesors, 33.2–36.6°C; amplitudes, 0.2–2.5°C; acrophases, 14:05–7:40), and Coretemp (mesors, 36.6–37.5°C; amplitudes, 0.2–0.7°C; bathyphases, 23:50–6:50). Higher PC loadings mainly corresponded to (i) large Chesttemp amplitudes, and phase advance of both temperature rhythms for the first PC (PC1, 27.2% of variance var.), (ii) high mesors in both temperature rhythms for PC2 (22.4% var.), and (iii) large Coretemp amplitudes for PC3 (12.9% var.). Chesttemp and Coretemp mesors and PC2 loadings decreased in females, while remaining quite stable in males as a function of age. In contrast, Coretemp amplitude and PC3 loadings increased with age in females, but decreased in males. Finally, older subjects, both female and male, displayed a reduction in ultradian variabilities, and an increase in both Chesttemp circadian amplitude and PC1 loadings.

**Interpretation:**

The dynamics relations between Chesttemp and Coretemp rhythms were largely moderated by age and sex, with results suggesting that treatment timing could be most critical for therapeutic index in women and in order people.

## Introduction

The circadian timing system (CTS) involves a complex network of molecular clocks that reside within each cell and are coordinated by a central pacemaker, the suprachiasmatic nuclei (SCN) in the hypothalamus (Hastings et al., 2003; Bass and Lazar, 2016; Panda, 2016). Molecular clocks involve over fifteen or more specific genes and regulate cellular metabolism and proliferation along the 24-h through the rhythmic control of transcriptional and posttranscriptional processes (Levi and Schibler, 2007; Takahashi, 2017). The SCN generate rhythmic neuroanatomic and humoral signals, which coordinate the cellular clocks along the 24-h timescale, and adjust their timing to environmental cycles (Levi and Schibler, 2007; Panda, 2016). In particular, the SCN generate the circadian rhythms in rest-activity and in body temperature, which have been proposed as CTS biomarkers (Ballesta et al., 2017).

Thus, circadian cycles in body temperature did entrain molecular clocks and clock-driven metabolism and cell cycle pathways in peripheral tissues or cultured cells (Brown et al., 2002; Buhr et al., 2010). Others and we have hypothesized earlier that their recording would convey critical information for the optimal timing of the delivery of medications (chronotherapy) in individual patients (Lévi et al., 2010; Tudela et al., 2010; Roche et al., 2014). Such personalized chronotherapy is needed for the optimization of treatment effects, because of large inter-subject differences in circadian rhythms from genes to biomarkers (Roche et al., 2014; Lévi et al., 2020). While the robustness of the 24-h patterns in rest-activity and cortisol secretion have been shown to serve as independent prognostic indicators of survival in cancer patients (Sephton et al., 2000; Lévi et al., 2014; Ballesta et al., 2017). The rhythm imposed by socio-professional routine is confounding the circadian timing information obtainable from rest-activity, as discussed in (Rietveld et al., 1993; Sephton et al., 2000), thus moderating its relevance to personalizing chronotherapy.

The critical importance of body temperature for health assessment was recently emphasized in a diverse cohort of 35,488 patients, where baseline body temperatures ranged from 35.3 to 37.7^*o*^C (Obermeyer et al., 2017). This inter-patient difference of up to 2.4^*o*^C was not explained by any infectious or inflammatory disease, with a limited influence of endocrine disorders (Obermeyer et al., 2017). In this large study performed in patients attending hospital clinics, body temperature was measured using an oral thermometer, without time of day specification. The most striking findings were that (i) measured factors explained only 8.2% of inter-subject temperature variation, and (ii) unexplained temperature variation was a significant predictor of subsequent mortality, with an increase of 0.149^*o*^C being significantly linked to a 8.4% higher 1-year mortality (Obermeyer et al., 2017). Thus, improving the precision of body temperature measurement and monitoring its circadian dynamics is potentially relevant for advancing human health and precision chronomedicine.

The circadian rhythm in core body temperature (Coretemp) results from the rhythmic control of heat gain and loss mechanisms by the SCN over the 24 h (Weinert and Waterhouse, 2007). In humans, heat gain mechanisms are usually at work in the morning and early afternoon, through nutritional and light intensity-related energy intake and adrenergic system-related vasoconstriction (Krauchi and Deboer, 2010). In contrast, heat loss mechanisms mostly involve skin surface vasodilation as a result of parasympathetic system activation, and predominate in the late afternoon and early night (Krauchi and Deboer, 2010). The relationship between core body and skin temperature has been further scrutinized and documented on limited time spans, i.e., 15-min (Lenhardt and Sessler, 2006) and 90-min (Eggenberger et al., 2018).

The assessment of circadian rhythms in body temperature could involve multiple daily measurements of rectal, vaginal, oral, or auricular temperature using conductive or contactless infrared thermometers or indwelling probes connected to wearable electronic devices (Longato et al., 2017). More recently, electronic gastro-intestinal pills have offered a novel means for minimally invasive continuous recording of Coretemp rhythms during real life (Travers et al., 2016; Fogt et al., 2017; Roxane et al., 2018; Komarzynski et al., 2019). The current main limitation of these temperature pills consists in their natural elimination into the feces within the 24–48 h that follow ingestion thus requiring a daily ingestion for two or more days in order to reliably document the circadian rhythms characteristics in an individual subject.

Skin temperature monitoring has been performed non-invasively using wired thermistor or thermocouples connected to wearable electronic devices, or, more recently, conductive wireless sensors that were patched or worn mostly at wrist or upper front lateral thorax (subclavicular) sites for days or weeks (Krauchi and Deboer, 2010; Tudela et al., 2010; Nicolas et al., 2013; Roche et al., 2014; Longato et al., 2017). In order to further integrate chest surface temperature rhythm (Chesttemp) parameters into daily precision chronomedicine, we have recently developed a mobile and comprehensive e-Health platform (Komarzynski et al., 2018). This platform comprises a chest activity and temperature sensor, which can tele-transmit minute measurements at the required frequency to a server, where multiple time series data analyses can be automatically performed, and potential decision-making information can be extracted (Huang et al., 2018; Komarzynski et al., 2018). Especially, our previous work based on the e-Health platform including 55 healthy individuals showed that around 24% of the subjects displayed a strong 12-h cyclicity in their Chesttemp rhythm and statistically significant sex- and age-related differences were identified in Chesttemp amplitude (Komarzynski et al., 2018).

In order to assess CTS functionality in temperature, we felt that it was critical to understand the relations between Chesttemp and Coretemp circadian rhythms, so as to effectively integrate chronotherapy concepts in daily medicine. Here, we measured both temperatures at chest surface and into the gut at the same times and under real life condition for at least 2 days (ST2). This has allowed us to reliably analyse their circadian oscillation. In addition, Chesttemp data from a study with a very similar design (ST1) were pooled (Komarzynski et al., 2018). This enabled us to further reveal age and sex trends on Chesttemp and Coretemp parameters and their mutual relations during daily life, and to highlight the influence of several other covariates, including chronotype, body mass index (BMI) and activity.

## Materials and Methods

### Study Designs and Data Collection

Common inclusion criteria comprised the ability to work or to perform usual activities, and to be aged 18 years or more. Non-inclusion criteria involved uncontrolled pathological or psychological conditions; any ongoing treatment with glucocorticosteroids, melatonin agonists or antagonists, lithium, or analgesic; any contraindication to the use of electronic devices; and night shift work or crossing of more than three time zones within the past 4 weeks. For ST2, any known gastrointestinal disease was also a non-inclusion criterion. ST1 was approved by the internal review boards at INSERM (Villejuif, France) and at Warwick University (Coventry, United Kingdom). ST2 was approved by the Ethical Committee of Warwick University (REGO-2017-2055). Both studies were conducted according to the Helsinki Declaration (Carlson et al., 2004). Subjects also provided signed informed consent forms before their participation.

Main characteristics of the subjects such as sex, age, weight, height, morningness–eveningness chronotype according to Horne and Östberg (1976), and concomitant medical condition and treatment were collected upon entry to both studies. The participants in both studies underwent remote monitoring of Chesttemp and activity for longer than 3 days using a wearable sensor attached to chest surface (Movisens, Karlsruhe, Germany) as part of the PicaDo mobile e-Health platform (Komarzynski et al., 2018). The chest sensor measured Chesttemp, accelerometry and 3-D orientation every minute and teletransmitted the measurements to a pocket-sized gateway (Eeleo, Montrouge, France) via Bluetooth Low Energy. The gateway teletransmitted the anonymized data via General Packet Radio Service (GPRS) to a secure and dedicated HL7-standards compliant server every 24 h. The participants in ST2 also had their Coretemp concurrently measured every minute via two ingestible electronic temperature pills (e-Celsius Performance Pill, BodyCAP Medical, Caen, France) swallowed 24-h apart by the subjects (Komarzynski et al., 2019). The gastrointestinal Coretemp data were transmitted via radio-frequency to a dedicated monitor. After both pills had been eliminated through the stools, data stored in the monitor were downloaded. Pseudonymized Chesttemp and Coretemp data were saved on a secure storage server according to the National Data Protection and Freedom of Information Acts guidance.

### Statistical Methods

#### Data Pre-processing

Non-physiological values in Chesttemp corresponded to sensor removal (typically for the purpose of showering) were identified as missing data by noting that the contemporaneous Chesttemp decreased to room temperature values (i.e., below 30°C). In some instances, the ingested pill was swallowed with a hot or a cold drink, which could sharply increase or decrease Coretemp values above 38°C or below 36°C within the initial 30 min following oral pill intake. These early Coretemp measures were identified by careful inspection of the time series, and were removed. The data from the first ingested pill were used up to the time point of its elimination in the feces after which the data from the second pill were used in order to produce a time-continuous Coretemp time series. The mean over 5-min intervals was computed for all temperature time series for noise and data size reduction. To perform cosinor regression modelling which aims at quantifying the parameters that summarised the averaged circadian cycle of temperature, the 5-min temperature data were further smoothed using a 1-h moving average window from which we computed the averaged 24-h day profile. Spectral analysis was applied to the hourly mean data which provides an adequate resolution of the periodogram at the circadian periods of interest (Costa et al., 2013). Missing hourly data points due to a sensor taken off for longer than 1 h were imputed via linear interpolation. For shorter removal times the available data within the hour was used to compute an hourly mean value. For gaps of missing data longer than 7 h, the recordings of the corresponding entire 24-h segment were omitted from the spectral analysis. This occurred once in Chesttemp for seven subjects.

#### Spectral Analysis

The spectral densities of both, Chesttemp and Coretemp, were estimated, along with their 90% confidence envelope, by applying Spectrum-Resampling (SR) algorithm (Costa et al., 2013) to the hourly means. The dominant period corresponding to the largest peak, as well as the gravity center of the spectrum (SGC) were identified. For the latter we computed $\text{SGC}≜\frac{\sum T_{k}\,I\,\left(T_{k}\right)}{\sum I\,\left(T_{k}\right)}$ where *I*(*T*_*k*_) is the estimated spectrum at period *T*_*k*_ for *T*_*k*_∈[2−h,54−h] and the summation is over *k* = 1–480^1^. The dominant period allowed us to classify all subjects into 24-h dominant, 12-h dominant and non-circadian groups. SGC summarized the importance of peaks over the spectra as a weighted average. We used time series data that covered at least three circadian cycles (i.e., 72-h) in order to obtain a suitably reliable spectral estimate of the circadian period lengths (Costa et al., 2013). Such recording durations were obtained for all 88 individual Chesttemp time series and for 16 out of 33 Coretemp time series.

#### Two-Harmonic Cosinor Regression

The aim of the cosinor regression modelling was to estimate and summarize the contribution of the two circadian frequencies, namely the 24- and 12-h periods, to the temporal dynamics of each temperature circadian oscillations. We hence fitted the following cosinor regression model (Cornelissen, 2014), with periods *T*_1_ = 12-h and *T*_2_ = 24-h to the averaged day profiles (time span from 12 p.m. to 11:59 a.m. clock time with 5-min resolution) of Chesttemp and Coretemp:

$$y\,\left(t\right)=M+a_{1}\,\sin\left(\frac{2\,\pi \,t}{T_{1}}\right)+b_{1}\,\cos\left(\frac{2\,\pi \,t}{T_{1}}\right)+a_{2}\,\sin\left(\frac{2\,\pi \,t}{T_{2}}\right)+b_{2}\,\cos\left(\frac{2\,\pi \,t}{T_{2}}\right)+e\,\left(t\right)$$

Where *y*(*t*) is the averaged temperature day profiles at time *t*; *M* is the mesor (mean level of the fitted cosine function); *a*_1_,*a*_2_and *b*_1_,*b*_2_are the coefficients of the cosinor model, and *e*(*t*) is a random quantity assumed to have zero mean. Given *T*_*1*_ and *T*_*2*_, the coefficients were estimated by linear least-squares regression. We hence collected the estimates of the five parameters {*M*,*a*_1_,*b*_1_,*a*_2_,*b*_2_} and the composite amplitude A, i.e., half range of the fitted values $\hat{y}\,\left(t\right)$. The acrophase for Chesttemp ø_max_, i.e., the time of the maximum of $\hat{y}\,\left(t\right)$, and the bathyphase of Coretemp ø_min_, i.e., the time of the minimum of $\hat{y}\,\left(t\right)$ were also computed. Note that bathyphase instead of acrophase was computed for Coretemp because it could be identified with a better precision than acrophase in most individuals (Komarzynski et al., 2019). Ninety percent confidence intervals for parameters were evaluated using the bootstrap method (Abdelhak and Iskander, 2004).

#### Principal Component Analysis

A principal component analysis (PCA) was applied to the pooled parameter estimates obtained from the cosinor regression models applied to Chesttemp and Coretemp thus characterizing the joint behavior of their circadian oscillations. Standard PCA was applied to the centered and scaled matrix, i.e., *Y* = [*y*_1_,*y*_2_,…,*y*_33_]′ ∈ *ℝ*^33×10^ where each row contains 10 parameter estimates *y*_*i*_ = [{*M*,*a*_1_,*b*_1_,*a*_2_,*b*_2_}_Chesttemp_,{*M*,*a*_1_,*b*_1_,*a*_2_,*b*_2_}_Coretemp_] ∈ *ℝ*^1×10^ corresponding to subject *i*, *I* = 1,…,33 in ST2. The resulting principal components (PCs) and their loadings describe orthogonal linear combinations in this 10-dimensional parameter space, ranked in importance (PC1, PC2, etc.) according to their contribution to explaining the total variability of the cosinor model parameter estimates across all individuals. We could then compute and plot the 2-dimensional (2-D) circadian oscillation pattern of Chesttemp and Coretemp, in the sequel referred to as “loop”, that was associated with the first three most highly ranked PCs.

#### Hidden Markov Model

A recently developed harmonic Hidden Markov Model (HMM) approach for accelerometer data was applied to quantify the individual daily activity strength (Huang et al., 2018). The HMM is a widely used statistical model which assumes that the observed time series data are a realization of a Markov process with unobserved states. In this study, the HMM approach was fitted to classify for each individual, retrospectively and probabilistically, the chest activity (concurrently measured with Chesttemp) into three states, namely inactive/rest state (IA), intermediately active state (MA), and highly active state (HA). The activity strength was evaluated as the median value of chest activity decoded as MA or HA states, i.e., intermediate-to-high activity.

#### Multivariate Regression Analysis With Covariates

Multivariate regression analysis allowed us to investigate the effect of covariates, such as sex and age, on other circadian parameters of interest, more precisely (1) the spectral gravity center (SGC) of Chesttemp; (2) the mesors and amplitudes of both Chesttemp and Coretemp; (3) the acrophase of Chesttemp and bathyphase of Coretemp; and (4) the first three principal components of matrix Y of estimated parameters from the cosinor regression model fitted to the Chesttemp and Coretemp time series. Note that two acrophases exist for subjects with 12-h domain period in Chesttemp and we used their evening acrophase that was consistently comparable to the expected acrophase of the subjects with 24-h period. Considering that phases are periodic along the 24-h timescale, acrophase and bathyphase were adjusted to linear values ranging from −10 (at clock hour, 14:00) to +10 (10:00). Let the estimates stated above under (1–4) represent the response variable, then the design matrix of the multivariate regression contained sex (*0* for females, 1 for males), age and a nonlinear interaction effect sex^∗^age as potential explanatory variables. We report the significance of their effect by means of the *p*-value of a two-sided *t*-test.

Spearman correlation and two-sample Welch’s *t*-test were applied to determine the potential relationship among circadian parameter estimates and subjects’ characteristics whenever applicable, and the significant results were reported here. Throughout the paper, statistical significance is considered for *p*-values below 5%, while a possible statistical trend was considered for *p*-values between 5 and 10%. All statistical analyses were performed in *R*.

## Results

### Participants’ Characteristics and Study Conduct

The pooled analysis study involved Chesttemp time series from 88 evaluated subjects, including 55 in ST1 and 33 in ST2. Overall, there were 38 males and 50 females, aged from 21 to 78 years, with approximately similar distributions across both STs (Table 1) regarding sex ratio (43.2% male), age (median, 35 years), weight (median, 71 kg), BMI (median, 24.3 kg/m^2^), intermediate-to-high daily activity (median of 107 accelerations/min), morningness or eveningness chronotype (30.7% morning type, 47.7% intermediate, and 5.7% evening type), and concomitant medical condition or medication intake. Nearly 70% of the subjects had no medical condition and were not taking any medication during their participation in either ST.

**TABLE 1:** Participants’ main characteristics.

Number of participants	ST1 55	ST2 33	Both 88
**Sex**			
M	23 (41.8)	15 (45.5)	38 (43.2)
F	32 (58.2)	18 (54.5)	50 (56.8)
**Age (years)**			
Mean ± SD^*a*^	38 ± 14	42 ± 18	39 ± 16
Median [1st, 3rd quartiles]	35 [27, 44]	35 [27, 53]	35 [27, 50]
Range	21–75	21–78	21–78
**Weight (kg)**			
Mean ± SD	69.6 ± 11.3	74.9 ± 15.4	71.6 ± 13.1
Median [1st, 3rd quartiles]	71.0 [62.5, 77.5]	71.0 [62.0, 89.0]	71.0 [61.9, 78.0]
Range	44.0–93.0	56.0–120.0	44.0–120.0
**Height (cm)**			
Mean ± SD	171 ± 9	172 ± 9	172 ± 9
Median [1st, 3rd quartiles]	171 [165, 174]	172 [167, 180]	172 [167, 178]
Range	155–195	152–188	152–195
Not available	28 (50.9)	0 (0)	28 (31.8)
**BMI (kg/m2)**			
Mean ± SD	24.0 ± 3.2	25.2 ± 4.8	24.7 ± 4.2
Median [1st, 3rd quartiles]	24.3 [22.3, 26.2]	24.4 [22.0, 27.2]	24.3 [22.0, 26.5]
Range	17.9–31.2	18.9–42.0	17.9–42.0
Not available	28 (50.9)	0 (0)	28 (31.8)
**BMI categories**			
Less than 18.5–underweight	1 (1.8)	0 (0)	1 (1.1)
18.5 to 24.9–healthy weight	15 (27.3)	18 (54.5)	33 (37.5)
25 to 29.9–overweight	10 (18.2)	13 (39.4)	23 (26.1)
30 to 39.9–obese	1 (1.8)	1 (3.0)	2 (2.3)
40 and over–morbidly obese	0 (0)	1 (3.0)	1 (1.1)
Not available	28 (50.9)	0 (0)	28 (31.8)
**Chronotype**			
Definite morning	2 (3.6)	5 (15.2)	7 (8.0)
Moderate morning	10 (18.2)	10 (30.3)	20 (22.7)
Intermediate	27 (49.1)	15 (45.5)	42 (47.7)
Moderate evening	2 (3.6)	3 (9.1)	5 (5.7)
Definite evening	0 (0)	0 (0)	0 (0)
Not available	14 (25.5)	0 (0)	14 (15.9)
**Chronotype score**			
Mean ± SD	54 ± 9	58 ± 12	56 ± 11
Median [1st, 3rd quartiles]	53 [47, 60]	57 [49, 67]	55 [47, 64]
Range	34–80	35–80	34–80
Not available	14 (25.5)	0 (0)	14 (15.9)
**Intermediate-to-high activity (accelerations/min)^*b*^**			
Mean ± SD	91.6 ± 47.6	109 ± 38.1	98.1 ± 45.2
Median [1st, 3rd quartiles]	102 (54.4, 123]	112 [75.2, 135]	107 [68.3, 129]
Range	17.2–194	43.5–196	17.2–196
**Concomitant medical condition**			
No	37 (67.3)	25 (75.8)	62 (70.5)
Yes	9 (16.4)	8 (24.2)	17 (19.3)
Not available	9 (16.4)	0 (0)	9 (10.2)
**Concomitant medication intake**			
No	35 (63.6)	25 (75.8)	60 (68.2)
Yes	11 (20.0)	8 (24.2)	19 (21.6)
Not available	9 (16.4)	0 (0)	9 (10.2)

*When not specified, the data are presented as Number of subjects (%). ^*a*^SD, standard deviation. ^*b*^Intermediate-to-high activity (accelerations/min) is estimated by the median value of the chest activity being decoded as intermediately active state or highly active state using Hidden Markov Model (Huang et al., 2018).*

**FIGURE 1:**
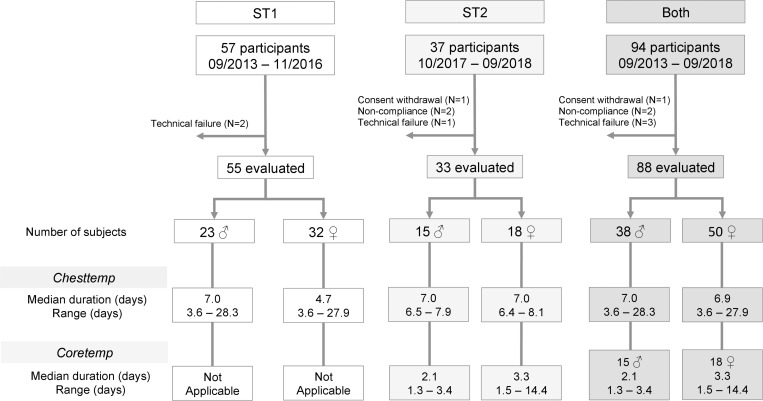
Consort diagram. Flow diagram showing the enrollment of subjects and the variables that were measured in each study, and for the current pooled analysis.

The median rate of missing temperature data was 4.0% [Interquartile range IQR 1.9–5.9%] out of the 88 Chesttemp records (extremes, none to 37.6%). Less than 0.2% of the data were missing in the 33 individual Coretemp records. The median duration of the Chesttemp time series was 7.0 days [IQR 6.8–7.3], with individual data lengths ranging from 3.6 to 28.3 days in both STs (Figure 1). For Coretemp time series in ST2, the median duration was 2.9 days [IQR 2.0–3.4], and ranged from 1.3 up to 14.4 days according to individual gastrointestinal transit (Figure 1). As a result, Coretemp time series exceeded 3 days for 16 subjects (48.5%). There was a trend toward a prolonged retention of the gastro-intestinal temperature pills in women as compared to men (two-sample Welch’s *t*-test, *p* = 0.002), as revealed with mean time series of 3.5 days as compared to 2.3 days, respectively.

### Inter-subject Difference of Circadian Rhythm

#### Results From Spectral Analysis

**FIGURE 2:**
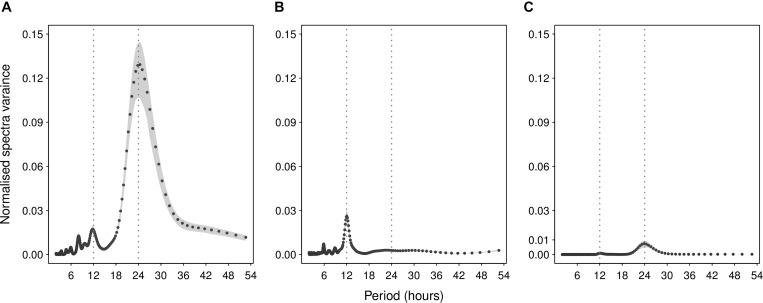
Illustrative examples of spectral analyses of Chesttemp or Coretemp time series. Plots of spectral density estimates (line) with respective 90% confidence intervals (gray area) for Chesttemp in panel **(A)** a 71 y.o female (subject A in ST2), and panel **(B)** a 34 y.o. male (subject B in ST2); and for Coretemp in panel **(C)** a 50 y.o. female (subject C in ST2). Corresponding dominant periods and SGC estimates (in hours) with 90% confidence interval were: **(A)** 24.4 h (23.3, 25.0) and 20.6 h (20.2, 20.9); **(B)** 12.0 h (11.9, 12.2) and 10.9 h (10.8, 11.2); **(C)** 23.8 h (23.3, 24.4) and 20.6 h (19. 9, 21.2). Note, for Chesttemp, higher spectral variance if largest peak near 24 h for panel **(A)** and smaller variance if largest peak near 12 h for panel **(B)**.

Circadian rhythm in Chesttemp was identified in 82 out of 88 subjects (93.1%), as revealed by the fact that 60 and 22 subjects had a dominant period around 24- or 12-h in ST1 and ST2, respectively (Figures 2A,B). Six subjects displayed no clear circadian pattern. The spectral gravity center (SGC), which summarised the spectral peaks as a weighted average, ranged from 10.3 to 22.0 h (median 15.1; IQR 13.0–17.6). Indeed, subjects with dominant 24-h period had higher SGC values than those with dominant 12-h period (one-tailed *t*-test *p* < 0.001). Thus, individuals with SGC values in the upper range had most spectral energy in the circadian domain, whilst those with SGC values in the lower range had 12-h dominant periods. The distributions for the 24- and 12-h dominant periods classification and SGC values were in good agreement in both STs (two-sample Welch’s *t*-test or Fisher’s exact test *p* > 0.1; Table 2).

**TABLE 2:** Circadian parameters distribution.

Number of participants	ST 1 55	ST 2 33	Both 88
**Parameter estimates obtained from spectral analysis**			
**Chesttemp**			
***Dominant Period^*a*^***			
24- ± 2-h ^	39 (71)	21 (68)	60 (69)
12- ± 1-h ^	13 (24)	9 (27)	22 (25)
***Gravity center of spectra (SGC)–hours***			
Mean (range)	15.2 (10.3–22.0)	15.4 (11.0–21.0)	15.3 (10.3–22.0)
Median [1st, 3rd quartiles]	14.6 [12.5, 15.2]	15.3 [13.2, 17.6]	15.1 [13.0, 17.6]
**Coretemp** (spectral analysis was applied to 16/33 participants in ST2)			
***Dominant Period^*a*^***			
24-h ± 2-h ^	–	16 (100)	–
12-h ± 1-h ^	–	0 (0)	–
***Gravity center of spectra (SGC)–hours***			
Mean (range)	–	19.8 (14.7–22.6)	–
Median [1st, 3rd quartiles]	–	20.1 [19.1, 20.7]	–
**Parameter estimates obtained from cosinor regression**			
**Chesttemp**			
***Amplitude–*°C**			
Mean (range)	0.87 (0.24–2.50)	0.89 (0.22–2.37)	0.88 (0.22–2.50)
Median [1st, 3rd quartiles]	0.78 [0.53, 1.11]	0.89 [0.57, 1.09]	0.81 [0.56, 1.10]
***Acrophase^*b*^–clock hours***			
Mean (range)	2:39 (14:05–7:40]	3:10 (22:30–7:40]	2:51 (14:05–7:40]
Median [1st, 3rd quartiles]	3:10 [2:12, 4:15]	3:00 [2:10, 3:55]	3:08 [2:10, 4:06]
***Mesor–*°C**			
Mean (range)	35.08 (33.19–36.60)	34.93 (33.85–36.63)	35.03 (33.19–36.63)
Median [1st, 3rd quartiles]	34.93 [34.64, 35.68]	34.81 [34.37, 35.46]	34.93 [34.49, 35.59]
**Coretemp**			
***Amplitude–*°C**			
Mean (range)	–	0.43 (0.17–0.70)	–
Median [1st, 3rd quartiles]	–	0.43 [0.35, 0.50]	–
***Bathyphase–clock hours***			
Mean (range)	–	3:32 (23:50–6:50]	–
Median [1st, 3rd quartiles]	–	3:30 [2:30, 4:15]	–
***Mesor–*°C**			
Mean (range)	–	36.99 (36.63–37.48)	–
Median [1st, 3rd quartiles]	–	36.95 [36.83, 37.11]	–

*Distributions of circadian parameter estimated by spectra analysis and two-harmonic cosinor regression modelling, for both Chesttemp (*N* = 88, ST1 and ST2) and Coretemp (*N* = 33, ST2). When not specified the data are presented as Number of subjects (%). ^*a*^Dominant periods were classified by 24-h or 12-h if their 90% confidence interval fall in the range of 24- ± 2-h and 12-h ± 1-h, respectively. ^*b*^Evening acrophase was reported for subjects with 12-h domain period in Chesttemp.*

Spectral analysis of the 16 subjects whose Coretemp records exceeded 3 days revealed a clear dominant spectral peak at 24-h (Figure 2C). Corresponding SGC ranged from 14.7 to 22.6 h (median 20.1; IQR 19.1–20.7).

#### Results From Cosinor Regression

Results from the cosinor regressions revealed relatively large inter-subject variations in the daily oscillation parameters of Chesttemp (Table 2). Chesttemp mesors and amplitudes ranged from 33.2 to 36.6°C (median 34.9; IQR 34.5–35.6) and from 0.22 to 2.5°C (median 0.81; IQR 0.56–1.1), respectively. The estimated composite amplitude of the (24- + 12-h) cosinor was positively correlated with the SGC obtained from spectral analysis (Spearman’s correlation *r* = 0.58, *p* < 0.001). As an example (Figures 2A,B) shows a much larger spectral power, SGC, and circadian amplitudes for subject A compared to subject B (both in ST2). The median Chesttemp acrophase was located at clock hour 03:08 [IQR 2:10–4:06], with individual values staggered over 17-h and 35 min. The Chesttemp parameters from cosinor analyses had a similar distribution in both STs (Table 2, two-sample Welch’s *t*-test *p* > 0.1).

**FIGURE 3:**
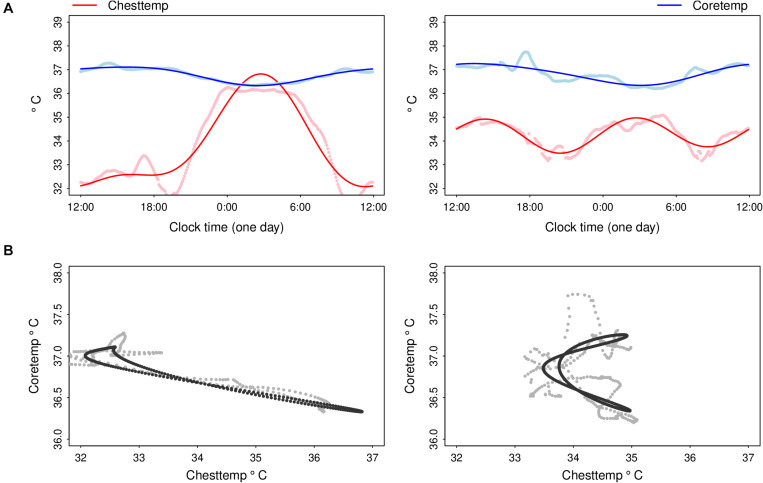
Illustrative examples of fitting composite circadian cosine regression of Chesttemp and Coretemp, and their reciprocal dynamics in the same subjects. **(A)** Averaged time series of Chesttemp and Coretemp over the 24-h time span (dots) for subjects A (left panel) and B (right panel) in ST2 (cf. Figure 2), and corresponding cosinor regression fittings with a composite period of (24- + 12-h) (solid lines) and **(B)** corresponding scatterplots of Chesttemp and Coretemp time series (dots) and cosinor regression fittings (solid lines). For subject A (ST2), mesors (M), amplitudes (Amp) and acrophase (ø_max_) or bathyphase (ø_min_), with their respective 90% confidence limits, were: M, 33.8^*o*^C (33.8, 33.9); Amp, 2.4^*o*^C (2.3, 2.5); ø_max_ (clock h), 2:45 (2:40, 2:50) for Chesttemp; and M, 36.8^*o*^C (36.8, 36.8); Amp, 0.4^*o*^C (0.4, 0.4); ø_min_, 2:30 (2:20, 2:35) for Coretemp. For subject B (ST2), Chesttemp parameter were M, 34.3^*o*^C (34.3, 34.30); Amp 0.7^*o*^C (0.7, 0. 8); ø_max_, 2:45 (2:40, 2:50) and 14:20 (14:15, 14:25); for Coretemp: M, 36.8 (36.8, 36.8); Amp, 0.5 (0.4, 0.5); ø_min_, 3:05 (2:45, 3:25). Note that an overall negative relation between Chesttemp and Coretemp time series was revealed in subject A (ST2), as illustrated with the folding of the joint cycle onto an almost straight line in 2-D thus indicating that both temperature time series were circadian rhythmic, and almost anti-phasic. For subject B (ST2), the relation between both temperature rhythms was best described with two loops over a 24-h span, i.e., a banana-shape folding of the joint oscillation in 2-D, as this subject had a 12-h dominant period in Chesttemp and a 24-h dominant period in Coretemp.

For the Coretemp rhythms, individual mesors ranged from 36.6 to 37.5°C, and individual amplitudes varied more than 4-fold, i.e., from 0.17 to 0.7°C (Figure 3A). The median bathyphase occurred at clock hour 03:30 [IQR 2:30–4:15], with individual values spread over a 7-h span. Statistically significant correlations were found between Coretemp and Chesttemp mesors (Spearman’s correlation *r* = 0.579, *p* < 0.001), and between their respective phases (*r* = 0.409, *p* = 0.018). No significant correlation was found between Chesttemp and Coretemp amplitudes and their corresponding SGC (*p* > 0.1).

One may ask the question why we performed both spectral analysis and cosinor regression. While spectral analysis focused on estimating the whole spectrum as a function of the range of frequencies to identify the dominant spectral peaks, the cosinor regression quantified the contribution of the two circadian harmonics, i.e., 12- and 24-h which were found to be dominant and (or) sub-dominant for most of the subjects, to the overall variation of the time series. Because cosinor analysis are with given harmonics and can thus be applied to shorter segments of data, in particular the 33 Coretemp time series. This then facilitates the further analysis (i.e., PCA) of the Chestemp and Coretemp oscillation loop.

### Oscillation Dynamics Linking Chesttemp and Coretemp

**FIGURE 4:**
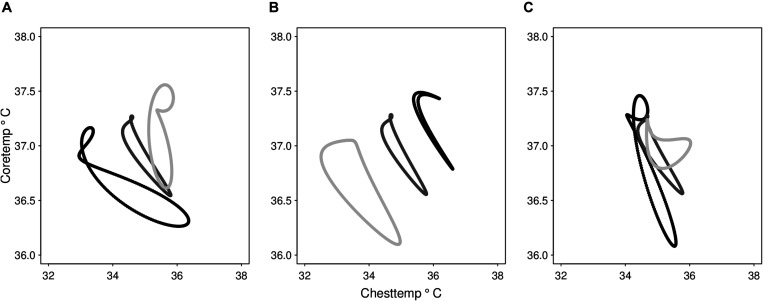
Illustrative examples of the main principal components reconstructions of the dynamics of Chesttemp and Coretemp circadian cycles. Reconstruction of the 24-h loop linking Chesttemp and Coretemp data based on single principal components (PC). For each PC, three subjects in ST2 were selected with corresponding PC values ranged from the highest (black), the median (dark grey), to the lowest (light grey). Reconstruction based on panel **(A)** PC1 that contains 27.2% of the variability (var.) of cosinor parameter set **Y**, **(B)** PC2 that contains 22.4% var., and **(C)** PC3 that contains 12.9 % var.

The circadian oscillation linking Chesttemp and Coretemp patterns, as predicted by the cosinor models, was visualized in 2-D for each of the 33 subjects in ST2 (Figure 3B and Supplementary Figure 1). The first three most highly ranked PCs, resulting from the PCA applied to the cosinor model parameter estimates, accounted for 27.2, 22.4, and 12.9% of the variance (var.), respectively, thus explaining 62.5% of the total var. of **Y.** Two-dimensional representations of the oscillation dynamics linking Chesttemp and Coretemp are shown as examples for three subjects, based on the respective contributions of PC1, PC2, and PC3 (Figures 4A–C). Thus, high loadings of PC1 corresponded to (i) large Chesttemp amplitudes (Spearman correlation *r* = 0.541, *p* = 0.001) and (ii) low Chesttemp and Coretemp mesors (*r* = −0.392, *p* = 0.025, and *r* = −0.445, *p* = 0.01, respectively). High values of PC2 values were indicative of (i) high Chesttemp and Coretemp mesors (*r* = 0.704 and *r* = 0.57, respectively, *p* < 0.001) and (ii) a 12-h rhythmic component in Chesttemp (associated with Chesttemp SGC with *r* = −0.317, *p* = 0.073); thus individuals with high PC2 values usually displayed a bimodal pattern in Chesttemp while those with high PC1 have a clear unimodal pattern. High PC3 values mainly captured large inter-subject variations in Coretemp amplitudes (*r* = 0.803, *p* < 0.001). Moreover, Spearman correlation revealed that high PC1 loadings were associated with an advanced phase in both Chesttemp (*r* = −0.504, *p* = 0.028) and Coretemp (*r* = −0.687, *p* = 0.001).

### Sex and Age Effects on Circadian Parameters

**FIGURE 5:**
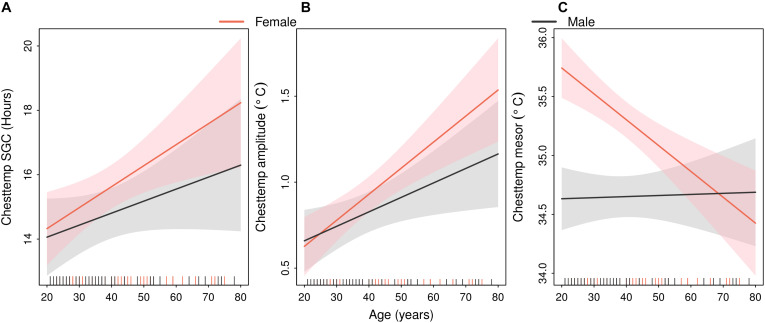
Sex-specific linear relations between age and Chesttemp circadian parameters. Estimated regression lines (solid lines) with 90% confidence bands (shaded areas) in males (black) or females (red). The vertical dashes in the abscissa indicate the age of each of the 88 subjects in both STs. **(A)** Chesttemp SGC, **(B)** Chesttemp amplitude, and **(C)** Chesttemp mesor.

Both the SGC and the circadian amplitude of Chesttemp increased with age, i.e., older people were more likely to display 24-h periodic oscillations with larger amplitudes as compared to younger ones (Figures 5A,B). No significant differences in either parameter were found according to sex. Coefficient estimates and *p*-values for the covariate sex, age and the interaction sex^∗^age are summarised in Table 3.

**TABLE 3:** Multivariate regression results.

Covariates	Sex^*a*^	Age (years)	Sex*age
**Response variables estimated from spectra analysis**			
**Chesttemp**			
***Gravity center of spectra (SGC)–hours***			
Coefficient estimates	0.294	0.065	−0.028
*p*-values	>0.1	0.022	>0.1
**Response variables estimated from composite cosinor model**			
**Chesttemp**			
***Amplitude–*°C**			
Coefficient estimates	0.167	0.015	−0.007
*p*-values	>0.1	<0.001	>0.1
***Acrophase*^*b,c*^*–clock hours***			
Coefficient estimates	−0.465	0.011	−0.007
*p*-values	>0.1	>0.1	>0.1
***Mesor–*°C**			
Coefficient estimates	−1.567	−0.022	0.023
*p*-values	<0.001	<0.001	0.01
**Coretemp**			
***Amplitude–*°C**			
Coefficient estimates	0.336	0.003	−0.008
*p*-values	0.002	0.062	0.001
***Bathyphase^*b*^–clock hours***			
Coefficient estimates	0.9	−0.019	0.002
*p*-values	>0.1	>0.1	>0.1
***Mesor–*°C**			
Coefficient estimates	−0.459	−0.006	0.006
*p*-values	0.007	0.018	0.074
**Response variables estimated from principal component analysis**			
**PC1 loading**			
Coefficient estimates	0.051	0.048	−0.002
*p*-values	>0.1	0.033	>0.1
**PC2 loading**			
Coefficient estimates	−3.722	−0.036	0.052
*p*-values	0.002	0.052	0.039
**PC3 loading**			
Coefficient estimates	1.713	0.024	−0.053
*p*-values	0.076	>0.1	0.014

*Coefficient estimates and *p*-values. ^*a*^Sex is encoded as 0 for females and 1 for males. ^*b*^Evening acrophase was used for subjects with 12-h domain period in Chesttemp. ^*c*^Acrophase and bathyphase were adjusted to linear values ranging from −10 (at clock hour, 14:00) to +10 (10:00).*

**FIGURE 6:**
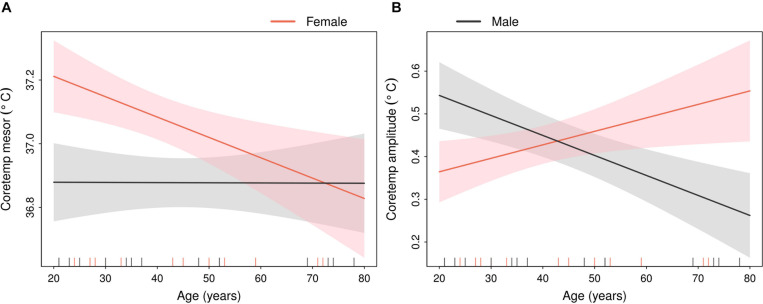
Sex-specific linear relations between age and Coretemp parameters from composite cosinor model. Estimated regression lines (solid lines) with 90% confidence bands (shaded areas) in males (black) or females (red). The vertical dashes in the abscissa indicate the age of each of the 33 subjects in ST2. **(A)** Coretemp mesor and **(B)** Coretemp amplitude.

On the other hand, the interaction between sex and age was found to have a significant effect on Chesttemp mesor, in each ST and in the pooled analysis (Figure 5C), in that Chesttemp mesors decreased with age in females, while they appeared unaffected by age in males. A similar sex-dependent effect of age was found for the Coretemp mesors (Figure 6A). This finding was consistent with the high correlation between Chesttemp and Coretemp mesors stated above. Furthermore, the Coretemp amplitudes increased with age in females, but decreased with age in males (Figure 6B). No significant sex and age effects were found for Coretemp bathyphase or Chesttemp acrophase (Supplementary Figure 2).

**FIGURE 7:**
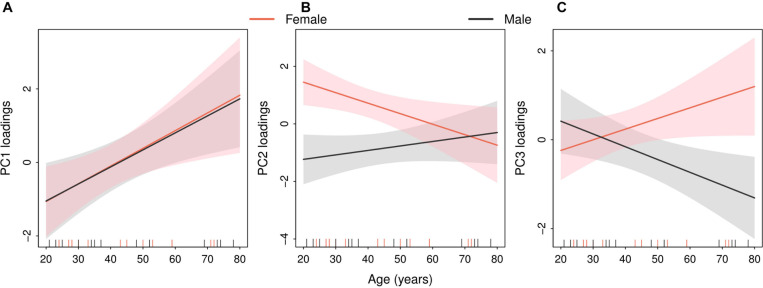
Sex-specific linear relations between age and the three main principal components linking the dynamics of Chesttemp and Coretemp rhythms. Estimated regression lines (solid lines) with 90% confidence bands (shaded areas) in males (black) or females (red). The vertical dashes in the abscissa indicate the age of each of the 33 subjects in ST2. **(A)** PC1 loadings, **(B)** PC2 loadings, and **(C)** PC3 loadings.

These results were consistent with the sex and age effects identified for the first three PCs (Figure 7). Thus, PC1 loadings increased with age both in females and in males (Figure 7A). PC2 loadings decreased in females and slightly increased in males with increasing age. Females younger than 50 years old showed higher values in PC2 loadings than males but the difference disappeared with aging (Figure 7B). Furthermore, an age and sex effect was found in PC3, whose loadings increased in females but decreased in males. A sex difference in PC3 loadings was mainly found in participants older than 50 years of age, as the sex-specific 90% confidence bands did not overlap (Figure 7C).

### Relations Between Circadian Parameters and Other Subjects’ Characteristics

Chesttemp mesors were negatively correlated with BMI (Spearman correlation, *r* = −0.338; *p* = 0.008). Both Chesttemp acrophase and Coretemp bathyphase were significantly correlated with the chronotype score (*r* = −0.511, *p* < 0.001 and *r* = −0.673, *p* < 0.001, respectively). This finding indicated that “morning chronotype” participants had phase advanced temperature rhythms as compared to the intermediate or evening chronotype. Such relation was further strengthened with the demonstration of a significant correlation between chronotype scores and PC1 loadings (*r* = 0.598, *p* = 0.002). High daily high-to-moderate activity levels were also found to be associated with low values in Chesttemp mesor (*r* = −0.216, *p* = 0.043), large circadian amplitudes in Chesttemp and Coretemp (*r* = 0.226, *p* = 0.034; and *r* = 0.355, *p* = 0.001, respectively), high values in PC3 loadings (*r* = 0.36, *p* = 0.04), and possibly high values in Chesttemp SGC (*r* = 0.195, *p* = 0.068).

Individuals with a concomitant medical condition had lower Coretemp amplitudes and PC3 values as compared to those without any (two-sample Welch’s *t*-test, *p* = 0.046 and 0.086, respectively). There was also a trend toward an earlier Chesttemp acrophase for subjects who took a medication (*p* = 0.083).

## Discussion

Large inter-subjects variations were found for the circadian rhythm parameters from telemonitored Chesttemp or Coretemp patterns in two studies conducted in the United Kingdom and in France. This was demonstrated (i) among the 94% of 88 people with a significant Chesttemp circadian rhythm that was telemonitored during their daily life for 3.6–28 days, and (ii) among the 33 people who also had their Coretemp concurrently telemonitored for 1.3–14.4 days, by means of two temperature electronic pills that were ingested 24 h apart. Individual subjects’ circadian mesor values varied over a range of 3.4^*o*^C for Chesttemp, and 0.9^*o*^C for Coretemp. These findings were in good agreement with the unexplained broad temperature variations that were prognostic of survival outcomes in a large cohort of patients (Obermeyer et al., 2017). In our studies, the circadian amplitudes further varied by nearly up to 11-fold for Chesttemp, and up to fourfold for Coretemp. The individual Chesttemp acrophases and Coretemp bathyphases were spread over a range of nearly 17 h and 7 h, respectively. Indeed, large inter-subjects variations have been reported for human circadian rhythms in body temperature and/or rest-activity (Tudela et al., 2010; Duffy et al., 2011; Roche et al., 2014; Mitchell et al., 2017), as well as for blood or urinary parameters (Lakatua et al., 1982; Ticher et al., 1994), despite similar light-dark and socio-professional synchronization. The inter-subject differences in circadian parameters were even more pronounced in cancer patients, whose treatment tolerability and overall survival related to sex-specific optimally timed chemotherapy (Lévi et al., 2007; Giacchetti et al., 2012; Innominato et al., 2020), indicating personalized treatment would be required.

Thus, it is critical to understand the relations between both body temperature rhythms in real life setting, in order to integrate circadian rhythm information within precision chronomedicine and personalized chronotherapy. Constant routine or forced desynchronization protocols allow for an apparent removal of masking environmental and lifestyle biases that enable the computation of the endogenous period, amplitude and bathyphase of the circadian rhythm in Coretemp, and that of melatonin secretion (Duffy et al., 2011; Phillips et al., 2019). For both of these rhythms sex was shown to play an important role on endogenous circadian period. Despite both of these rhythms are established markers of the endogenous circadian phase, their parameters displayed large inter-subject variations (Duffy et al., 2011). The masking effects of the individual subject’s responses to the severe constraints and stress that may be generated by these protocols have indeed been seldom considered (Rietveld et al., 1993). Moreover, such protocols could hardly be implemented into chronomedicine, given the need for minimally invasive and time-consuming procedures, for instance in order to determine optimal treatment timing in patients.

The complex effects of aging and physical activity on circadian rhythms in body temperature have been addressed in several reviews (Weinert and Waterhouse, 2007; Batinga et al., 2015). In the present pooled studies (ST1 and ST2), older people consistently displayed less ultradian variabilities and larger circadian amplitudes of Chestemp, as compared to younger ones. Such age-related trends were more prominent in females than in males. Thus, the results somewhat differed from those in an earlier report involving a smaller sample size (*N* = 55 from ST1), where males showed a slightly decreasing trend as a function of age (Komarzynski et al., 2018). Here, the Coretemp amplitude also increased in older women as compared to younger ones, yet it decreased in older men as compared to younger ones. Such sex-age related interactions deserve confirmation in a larger population, since it was shown here for 33 subjects. Nevertheless the *a priori* sex and age stratification in ST2 strongly supports the reliability of the current findings. Principle component analyses further highlighted sex-age interaction on the relations between both temperature rhythms.

Among the main influential factors, we identified the level of intermediate-to-high activity, as modeled according to Hidden Markov Model, as a determinant of the circadian amplitude in both temperature rhythms. This was in good agreement with prior reports where this issue was addressed using different methods (Waterhouse et al., 2005). Intermediate-to-high activity was not influential on the detection of the dominant period length in Chesttemp (i.e., 24- vs. 12-h). However, there was a possible significant trend toward an association of intermediate-to-high activity with Chesttemp SGC (Spearman correlation, 0.05 < *p* < 0.1), thus supporting a possible influence on the overall period spectrum. There was no evidence of correlation between intermediate-to-high activity with age or sex. Also, “morning chronotype” participants had phase advanced chest and core temperature rhythms as compared to the intermediate or evening chronotype, in good agreement with a prior report (Martinez-Nicolas et al., 2019). In another recent report, we have developed a model for the prediction of individual Coretemp bathyphase, based on sex, rest-activity center of rest time, Chestemp rhythm acrophase and chronotype (Komarzynski et al., 2019).

In conclusion, this work highlights the need for modelling Chesttemp rhythm outputs in order to gain proper insight into Coretemp rhythm parameters, which may be critical for optimal treatment timing. The sensor-gateway-analysis platform we used here was part of a more comprehensive domomedicine platform which is now entering clinical trial testing in pancreatic cancer patients (Bouchahda et al., 2020). Several additional steps will be needed in order to use such information for the personalization of cancer chronotherapy. At a preclinical level, chronotherapeutic algorithms have begun to be developed from synchronized cell cultures to rodent models (Dulong et al., 2015; Ballesta et al., 2017). Such chronotherapeutic algorithms can be linked to temperature cycles (Abraham et al., 2019), be moderated according to sex and aging (Martinelli et al., 2021) and tentatively upscaled to humans (Ballesta et al., 2017). Translational and clinical trials will then be needed to validate the clinical relevance of personalized treatment timing based on both Chesttemp and rest-activity telemonitoring. Our ultimate goal involves the delivery of real time information on circadian rhythms of individual patients to the medical team in order both to detect early improvements or deterioration in patients, and to compute optimal treatment times for individual patients, based on these circadian biomarkers.

## Data Availability Statement

The raw data supporting the conclusions of this article will be made available by the authors, without undue reservation.

## Ethics Statement

The studies involving human participants were reviewed and approved. ST1 was approved by the internal review boards at INSERM (Villejuif, France) and at Warwick University (Coventry, United Kingdom). ST2 was approved by the Ethical Committee of Warwick University (REGO-2017-2055). Both studies were conducted according to the Helsinki Declaration (Carlson et al., 2004). The participants provided their written informed consent to participate in the studies.

## Author Contributions

QH designed, analysed, and interpreted this study, and wrote the manuscript. SK, MB, BF, and FL designed, conducted, analysed and summarised ST1 and ST2, helped interpret the pooled studies, and contributed to the writing of the manuscript. All authors contributed to the article and approved the submitted version.

## Conflict of Interest

The authors declare that the research was conducted in the absence of any commercial or financial relationships that could be construed as a potential conflict of interest.

## Footnotes

1 Zero padding (Percival and Walden, 2020) was applied to the time series (to increase the size to 1,000) to obtain a smoother representation of the estimated spectrum before the Discrete Fourier Transform was computed. We focus on the estimated spectrum with periods ranging from 2- to 54-h containing 480 period bins in total, which are adequate to cover the circadian periods of interest.
