# Supplementary material for: Telemonitored Human Circadian Temperature Dynamics During Daily Routine
Source: Front Physiol. 2021 May 10;12:659973. doi: 10.3389/fphys.2021.659973 (PMC8141869; doi:10.3389/fphys.2021.659973)
Supplement: Supplementary file 1 [file Data_Sheet_1.docx]

**Supplementary Figure 1 Scatterplots of Chestemp (x-axis) and Coretemp (y-axis) time series (dots) and cosine functions (solid lines), for all 33 subjects in ST2.**

(**A**) Subject id from 1 to 20; (**B**) Subject id from 21 to 33. *Note that id has been re-numbered to maintain anonymity. Example subjects A, B and C presented in* ***Figure 2,3*** *in the main manuscript were marked.*

**Supplementary Figure 2 Sex-specific linear relations between age and the circadian timing of both Chesttemp and Coretemp rhythms.**

Estimated regression lines (solid lines) with 90% confidence bands (shaded areas) in males (black) or females (red). The vertical dashes in the abscissa indicate the age of each of 87 subjects (both STs) for Chesttemp acrophase (**A**), and that of each of 33 subjects (ST2) for Coretemp bathyphase (**B**). P-values > 0.1 for male sex, age and male sex*age. *One outlier sample of Chestemp acrophase (located at clock hour 14:05) was removed in the multivariate regression analysis.*
