# Supplementary figures and images for: Telemonitored Human Circadian Temperature Dynamics During Daily Routine
Source: Front Physiol. 2021 May 10;12:659973. doi: 10.3389/fphys.2021.659973 (PMC8141869; doi:10.3389/fphys.2021.659973)

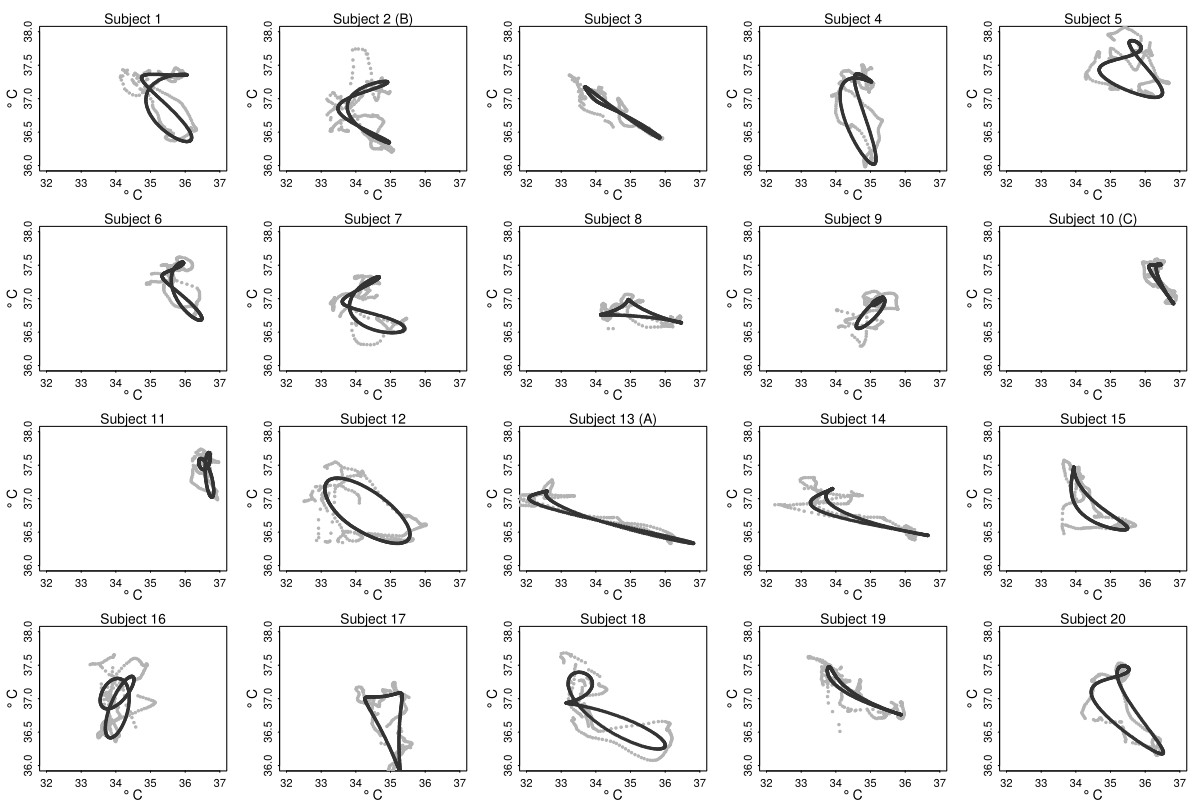

Supplement: Supplementary file 2 [file Image_1.TIF]

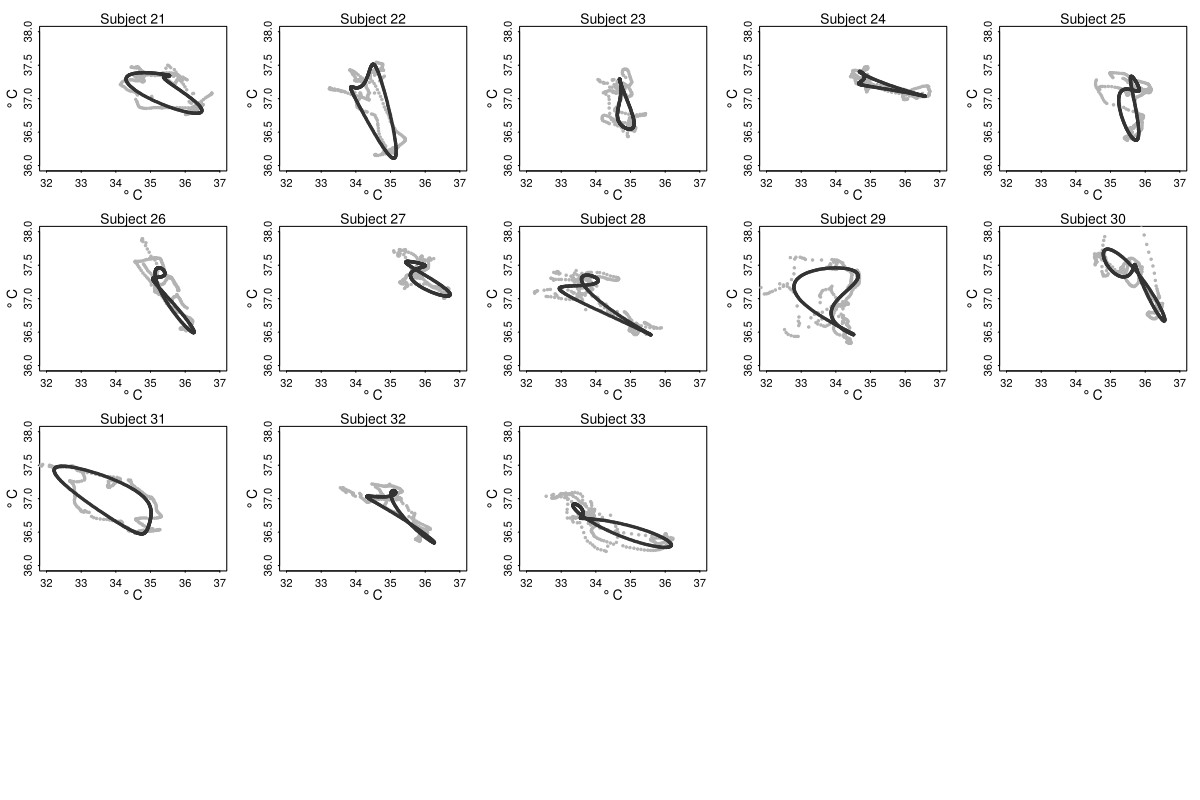

Supplement: Supplementary file 3 [file Image_2.TIF]

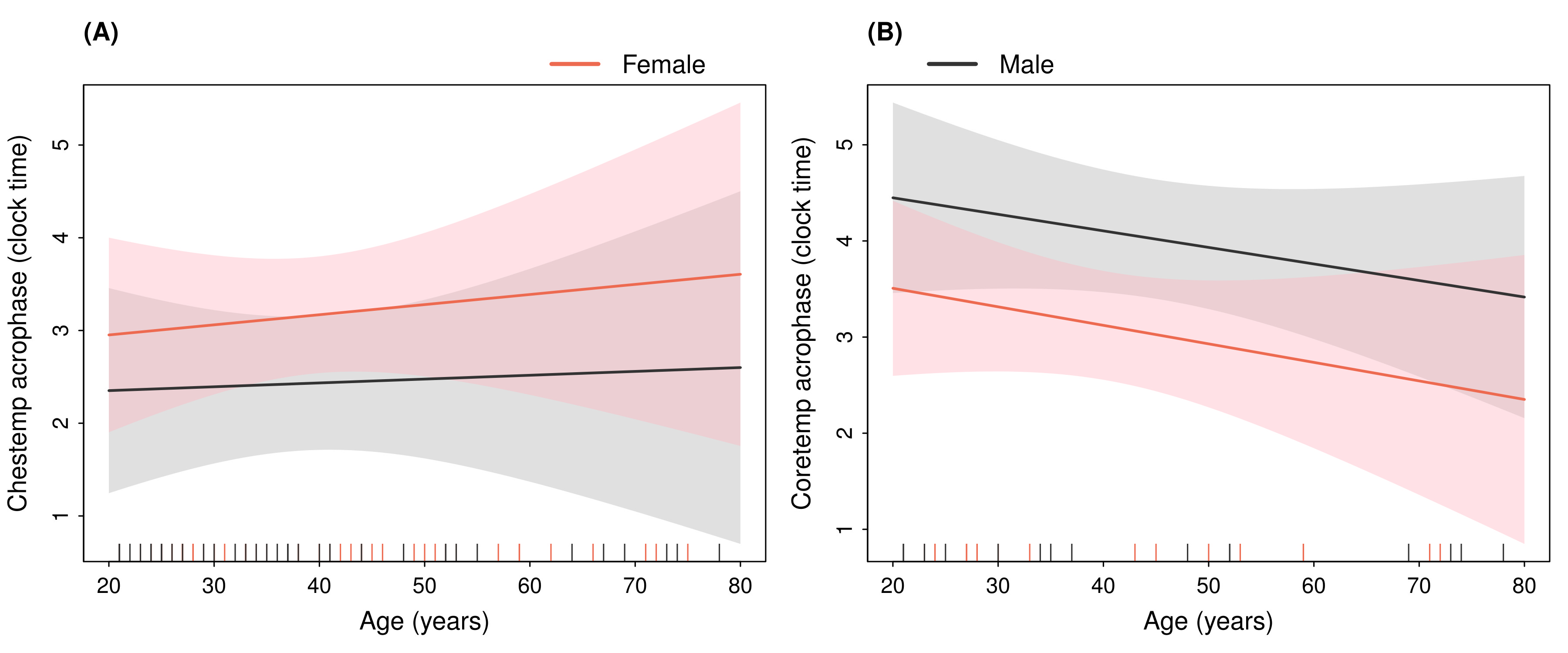

Supplement: Supplementary file 4 [file Image_3.TIF]
